# Obesity-Activated Lung Stromal Cells Promote Myeloid Lineage Cell Accumulation and Breast Cancer Metastasis

**DOI:** 10.3390/cancers13051005

**Published:** 2021-02-28

**Authors:** Lauren E. Hillers-Ziemer, Abbey E. Williams, Amanda Janquart, Caitlin Grogan, Victoria Thompson, Adriana Sanchez, Lisa M. Arendt

**Affiliations:** 1 Program in Cellular and Molecular Biology, University of Wisconsin-Madison, Madison, WI 53706, USA; lauren.ziemer@nih.gov; 2 Program in Comparative Biomedical Sciences, University of Wisconsin-Madison, Madison, WI 53706, USA; awilliams33@wisc.edu; 3 Department of Comparative Biosciences, University of Wisconsin-Madison, Madison, WI 53706, USA; ajanquart@mcw.edu (A.J.); cgrogan62@midwestern.edu (C.G.); victoria.thompson@wisc.edu (V.T.); asanchez29@wisc.edu (A.S.)

**Keywords:** obesity, breast cancer, metastasis, macrophages, collagen, lung fibroblasts

## Abstract

**Simple Summary:**

Obese breast cancer patients have an increased risk for metastasis; however, the mechanisms are not well characterized. Here, we show that obesity promotes rapid mammary tumor growth and enhances metastases in mice. Within the lungs of non-tumor-bearing mice, obesity increased recruitment of myeloid lineage cells and elevated deposition of collagen fibers. Lung stromal cells isolated from obese mice demonstrated increased expression of extracellular matrix proteins and inflammatory cytokines, including colony-stimulating factor 2 (CSF2). Conditioned media from lung stromal cells of obese mice promoted the invasion of myeloid lineage cells, which was dependent upon lung stromal cell expression of CSF2. These studies suggest that obesity creates an environment conducive to metastatic growth in the lungs prior to tumor formation. Understanding how obesity promotes metastases may increase therapeutic options for a growing population of obese breast cancer patients.

**Abstract:**

Obesity is correlated with increased incidence of breast cancer metastasis; however, the mechanisms underlying how obesity promotes metastasis are unclear. In a diet-induced obese mouse model, obesity enhanced lung metastasis in both the presence and absence of primary mammary tumors and increased recruitment of myeloid lineage cells into the lungs. In the absence of tumors, obese mice demonstrated increased numbers of myeloid lineage cells and elevated collagen fibers within the lung stroma, reminiscent of premetastatic niches formed by primary tumors. Lung stromal cells isolated from obese tumor-naïve mice showed increased proliferation, contractility, and expression of extracellular matrix, inflammatory markers and transforming growth factor beta-1 (TGFβ1). Conditioned media from lung stromal cells from obese mice promoted myeloid lineage cell migration in vitro in response to colony-stimulating factor 2 (CSF2) expression and enhanced invasion of tumor cells. Together, these results suggest that prior to tumor formation, obesity alters the lung microenvironment, creating niches conducive to metastatic growth.

## 1. Introduction

Global obesity rates, as defined by a body mass index (BMI) greater than 30.0 kg/m^2^, have nearly tripled since 1975; approximately 13% of the world’s population is considered to be obese, including 15% of women [1]. Obesity increases the risk of breast cancer in postmenopausal women, as well as premenopausal women who have elevated risk due to heritable factors [2,3,4]. Regardless of menopausal status, obese breast cancer patients have an enhanced risk of developing distant metastases compared to lean patients [5,6], particularly to the liver and lungs [7]. While the five-year survival rate of metastatic breast cancer patients has significantly increased over the last 30 years [8], metastasis accounts for the vast majority of breast cancer-related deaths. The mechanisms of how obesity promotes metastatic breast cancer are largely unknown. As obese patients are also at an elevated risk for treatment resistance [9,10,11], understanding the relationship between obesity and metastasis is vital to develop targeted therapies for obese patients. 

Metastasis is a complex process in which tumor cells escape the primary tumor, survive in circulation, extravasate at distal sites, and proliferate in competent organs. Evidence from preclinical models has suggested that primary breast tumors secrete factors and exosomes which promote metastasis through establishment of premetastatic niches in potential metastatic organs [12,13]. A major component of premetastatic niches are bone marrow-derived myeloid lineage cells, including monocytes, macrophages, neutrophils and myeloid-derived suppressor cells (MDSCs). MDSCs are a heterogeneous population of CD11b^+^ myeloid cells classified into two subsets: granulocytic MDSCs (gMDSCs), most similar to neutrophils, and monocytic MDSCs (mMDSCs), which resemble monocytes. Expansion of MDSC subtypes is dependent on systemic and microenvironmental cues [14,15], as MDSCs are absent in healthy individuals [16] but increase under conditions of obesity [17,18,19]. Myeloid lineage cells are thought to aid in the establishment of an environment conducive to metastatic growth through secretion of cytokines, extracellular matrix (ECM) remodeling, and immunosuppression [12,20]. Although obesity results in recruitment of myeloid lineage cells into obese adipose tissue [21], little is known regarding the effects of obesity on the immune populations in distant sites which might contribute to metastasis. 

Within the premetastatic environment, tumor-secreted factors alter stromal cells, leading to changes in expression of ECM proteins and matrix metalloproteinases [12,22]. Studies have suggested that successful metastatic colonization occurs through both structural alterations of the ECM and deposition of new ECM components within premetastatic niches [13,23]. Stromal cells secrete ECM proteins, such as fibronectin, which facilitate tumor cell adhesion and colonization [24]. Lung stromal cell activation has been observed in other pathological conditions, such as idiopathic pulmonary fibrosis, and increased immune cells and serum cytokines have been shown to play a role [25,26]. Obesity leads to chronic inflammation within adipose tissue, resulting in increased circulating levels of multiple inflammatory cytokines [27,28]. Within the mammary gland, obesity activates adipose-derived stromal cells, promoting mammary tumor progression [29]. However, how obesity impacts stromal cells at distant sites has not been examined, and these changes may significantly enhance distal metastatic colonization. 

Here, we examined how obesity promotes breast cancer metastasis through activation of the lung microenvironment. We show that obesity enhances metastasis to the lungs both in the presence and absence of primary mammary tumors. Lungs from obese mice demonstrated increased recruitment of myeloid lineage cells prior to and during metastatic growth. In the absence of primary tumors, lung stromal cells isolated from obese mice demonstrated increased proliferation rates, enhanced collagen deposition, and elevated expression of proinflammatory cytokines compared to lung stromal cells from lean mice. Further, conditioned media from lung stromal cells from obese mice enhanced invasion of bone marrow-derived myeloid lineage cells in culture through elevated expression of colony-stimulating factor 2 (CSF2). Overall, our findings suggest that obesity activates lung stromal cells prior to tumor formation, leading to increased myeloid lineage cell recruitment, similar to premetastatic niche formation by tumor cell-secreted factors during cancer progression. These changes in the lung microenvironment in obesity may contribute to the increased metastatic incidence observed in obese breast cancer patients, as well as other obesity-related cancers. 

## 2. Materials and Methods

### 2.1. Animal Studies

All procedures involving animals were approved by the University of Wisconsin-Madison Institutional Animal Care and Use Committee (Animal Welfare Assurance Number (D16-00239)). Female FVB/NTac mice were purchased from Taconic Laboratories and maintained according to the Guide for Care and Use of Laboratory Animals in AAALAC-accredited facilities. Three-week-old FVB/N female mice were randomized to be fed a low-fat diet (LFD, 10% kcal from fat, Test Diet; 58Y2) or high-fat diet (HFD, 60% kcal from fat, Test Diet; 58Y1) for 16 weeks to induce obesity. Purified diets contained equal amounts of vitamins and micronutrients. Body mass was measured weekly. 

### 2.2. Cell Lines

Met-1 cells were provided by Dr. Alexander Borowsky [30] and were transduced with lentivirus encoding green fluorescent protein (GFP) as described [31]. TC2 GFP^+^ cells were provided by Dr. Linda Schuler [32]. Primary lung stromal cells were isolated from lungs of LFD- and HFD-fed mice. Lung tissue was digested for 1 h in DMEM:F12 (Corning; 10-090-CV, Corning, NY, USA) supplemented with 3 mg/mL collagenase I (MilliporeSigma; 1148089, Burlington, MA, USA). Digested tissue was incubated for 2 h to collect adherent cells, and adherent cells were expanded in culture for no more than three passages prior to use in assays. Met-1 tumor cells were cultured in DMEM (Corning; 10-017-CV, Corning, NY, USA) supplemented with 10% FBS, lung stromal cells were cultured in DMEM supplemented with 10% calf serum, and TC2 cells were cultured in DMEM supplemented with 10% FBS and 1 mg/mL G418 (ThermoFisher Scientific; 11811023, Waltham, MA, USA). All media contained 1% antibiotic/antimycotic solution, and cells were maintained at 37 °C in 5% CO_2_. Tumor cell lines were not further validated and were tested for mycoplasma prior to use in experiments (Idexx BioAnalytics, Columbia, MO, USA). 

### 2.3. Tumor Cell Transplantations 

To generate tumors, 5 × 10^5^ Met-1 or 2.5 × 10^4^ TC2 cells were suspended in 2:1 Matrigel:DMEM (for the former: Corning; 354234, Corning, NY, U.S.A.) and injected bilaterally into the inguinal mammary glands of LFD- or HFD-fed FVB/N female mice. Tumor diameters were measured three times each week using calipers. Tumor volume was calculated using the formula 4/3πr^3^. To generate metastases, 5 × 10^5^ Met-1 or TC2 cells were suspended in sterile PBS and injected into the tail vein of LFD- or HFD-fed mice. End stage for metastatic development following tail vein injection of tumor cells was defined as 6 weeks for mice transplanted with Met-1 cells or 8-weeks for TC2 recipient mice. 

### 2.4. Conditioned Media and Invasion Assays

Lung stromal cells were grown on 100 mm plates until confluent. Cells were washed with PBS and then grown for 24 h in DMEM supplemented with 0.5% calf serum and 1% antibiotic/antimycotic solution. Conditioned media were filtered through 0.22 μm filters (ThermoFisher Scientific; 09-720-004, Waltham, MA, USA). To assess invasion, 1 × 10^5^ bone marrow cells from LFD-fed mice were plated in duplicate in serum-free media on inserts with 8 µm pores (Corning; 353097, Corning, NY, USA) coated with 1 mg/mL Type I rat tail collagen (Corning; 354236, Corning, NY, USA), and invasion toward conditioned media from lung stromal cells was measured after 4 h. Inserts were formalin-fixed and stained with 0.1% crystal violet. Four images of each invasion insert were taken at 100× magnification on a Nikon Eclipse E600 Microscope with a QICAM Fast 1394 camera and quantified using ImageJ (NIH, RRID:SCR_003070) with cell counter plug-in. Bone marrow cells that invaded into the conditioned media were quantified using a hemocytometer, then cytospun onto slides, fixed in methanol and stained using antibodies for CD45 (ThermoFisher Scientific; 14-0451-82, Waltham, MA, USA), CD11b (Novus Biologicals; NB110-89474, Centennial, CO, USA), and Ly6G (Abcam; ab25377, Cambridge, MA, USA). Invasion was also quantified in response to serum-free DMEM supplemented with 10 ng/mL of recombinant mouse CSF2 (R&D Systems, 415-ML-5, Minnesota, MN, USA), PBS, or conditioned media from lung stromal cells from HFD-fed mice treated with 5 µg/mL of either CSF2 neutralizing antibodies (R&D Systems, MAB415-100, Minnesota, MN, USA) or rat IgG antibodies (R&D Systems, 6-001-A, Minnesota, MN, USA) for 1 hr prior to the assay start. Conditioned media were collected from lung stromal cells treated with serum from HFD-fed mice supplemented with 10 µM of transforming growth factor beta (TGFβ) inhibitor SB431542 (Adooq Biosciences, A10826A, Irvine, CA, USA) or DMSO control for 6 days. Treated cells were washed with PBS and then conditioned media were collected in serum-free DMEM for 24 h. Four biological replicates were tested for each condition.

### 2.5. Quantification of Changes in Cell Number

To quantify changes in cell number, 1 × 10^5^ lung stromal cells were plated in DMEM supplemented with 10% calf serum and 1% antibiotic/antimycotic solution. For serum treatment experiments, 1 × 10^5^ lung stromal cells from LFD-fed mice were plated with DMEM+5% serum collected from LFD- or HFD-fed mice+1% antibiotic/antimycotic solution. To test responses to TGFβ1, lung stromal cells from LFD-fed mice were grown in 5% serum from LFD- or HFD-fed mice supplemented with 10 µM of SB431542 or DMSO control or in DMEM+0.5% calf serum supplemented with 5 ng/mL recombinant mouse TGFβ1 (Bio-Techne Corporation, 7666-MB, Minneapolis, MN, USA) or PBS vehicle control. Cells were fed with media supplemented with serum every 2 days. All cell number assays were plated in triplicate and counted after 6 days with a hemocytometer and then pelleted for RNA extraction. Latent TGFβ1 was activated prior to quantification, and total TGFβ1 was measured in serum from LFD- and HFD-fed mice diluted 1:50 in buffer using TGFβ1 DuoSet ELISA (Bio-Techne Corporation, DY1679, Minneapolis, MN, USA) in duplicate per the manufacturer’s instructions. 

### 2.6. Contractility Assays

Type I rat tail collagen (Corning, 354236, Corning, NY, USA) was diluted and neutralized in an equal volume of filter sterilized HEPES. In total, 5 × 10^4^ lung stromal cells were added for a final concentration of 2 mg/mL collagen. The neutralized collagen and cell mixture were plated on 6-well plates in triplicate and incubated at 37 °C with 4 biological replicates. After 4 hr, the gels were released and floated in 2 mL of DMEM supplemented with 10% calf serum and 1% antibiotic/antimycotic solution. The gel diameter was measured with a ruler at days 0, 2, 4, 5, and 7. Gels were fed after measurement on days 2 and 4. Contracted area was calculated using A = πr^2^ by subtracting the area measured on day 7 from day 0. On day 7, gels were digested with collagenase for 10 min at 37 °C, and the difference in area was divided by the number of cells in the gel at day 7.

### 2.7. Quantitative RT-PCR

RNA was isolated from lung stromal cells using TRIzol (Life Technologies, 15596026, Calsbad, CA, USA) and purified using Qiagen RNeasy Mini Kit (Qiagen; 74104, Germantown, MD, USA). RNA was reverse transcribed using the High Capacity cDNA Reverse Transcription Kit (Applied Biosciences, 4368814, Beverly Hills, CA, USA) and Techne Thermal Cycler. Quantitative RT-PCR was performed using iTaq SYBR Green Supermix (Bio-Rad, 172-5121, Hercules, CA, USA) with a Bio-Rad CFX Connect Real-Time PCR Detection System (Bio-Rad, Hercules, CA, USA). Transcripts were normalized to housekeeping gene hypoxanthine-guanine phosphoribosyltransferase (Hprt) and data were analyzed using the ∆∆Cq method (fold change) or ∆Cq method (relative expression). Primer sequences are listed in Table S1.

### 2.8. Flow Cytometry

Lung tissue was dissociated to single cells and resuspended at 1 × 10^6^ cells/mL in PBS containing 1% calf serum. Dissociated lung cells were blocked with CD16/32 antibodies (ThermoFisher Scientific; 14-0161-82, Waltham, MA, USA) for 30 min at 4 °C. Cells were stained with fixable viability dye eFluor 780 (ThermoFisher Scientific; 65-0865-14, Waltham, MA, USA) per manufacturers’ instructions, then incubated with antibodies to detect CD45-PE (0.5 μg/μL), CD11b-APC (0.5 μg/μL), Ly6C-eFluor 700 (1.5 μg/μL), Ly6G-BV421 (1 μg/μL), and CD115-PE-Dazzle594 (1 μg/μL) for 30 min at 4 °C. Antibody-bound cells were resuspended at 1 × 10^6^ cells/mL and quantified using an Attune Nxt Flow Cytometer (ThermoFisher Scientific, Waltham, MA, USA). Flow cytometry data were analyzed with the FlowJo software package v 10 (FlowJo, LLC, RRID:SCR_008520). Gates were set on fluorescence minus one controls and analyzed according to published guidelines [33]. All antibody source numbers and clones are listed in Table S2.

### 2.9. Histology and Immunofluorescence

Paraffin-embedded lungs were sectioned and stained with Hematoxylin and Eosin (H&E) by the Experimental Pathology Laboratory (Carbone Cancer Center, University of Wisconsin-Madison, Madison, WI, USA). Tissue staining for estrogen receptor alpha (ERα), F4/80, CD11b, Ly6G, and GFP was performed as described [34]. All antibody source numbers and clones are listed in Table S2. CD11b^+^, F4/80^+^, and Ly6G^+^ cells were quantified in nonmetastatic and metastatic lungs using ImageJ (NIH, RRID:SCR_003070) and divided by either the total lung tissue area on each image or by the metastatic area on the image. Blinded tissue sections were imaged using a Nikon Eclipse E600 Microscope (RRID:SCR_018858) and QICAM Fast 1394 camera (Teledyne Photometrics, Tuscon, AZ, USA). Five images were taken for each lung and quantified from 5–9 lungs/group. To quantify metastases, clusters of 5 or more GFP^+^ Met-1 or TC2 tumor cells were considered as metastatic lesions. 

### 2.10. Collagen Quantification

Paraffin-embedded lung sections from LFD- and HFD-fed mice were deparaffinized and rehydrated through alcohols. Slides were incubated for 1 hr in picrosirius red solution (0.5 g of Direct Red 80 (Sigma-Aldrich; 2610-10-8, St. Louis, MO, USA) in 500 mL of saturated picric acid (Sigma-Aldrich; P6744-1GA, St. Louis, MO, USA)). Slides were washed twice with acidified water (0.5% acetic acid) for 10 min, dehydrated in graded ethanol and xylenes, and mounted using Richard-Allan mounting medium (ThermoFisher Scientific, 4112APG, Waltham, MA, USA). Imaging of picrosirius red was performed using a Nikon Eclipse E600 Microscope and QICAM Fast 1394 camera. Collagen fluorescence was detected using a TRITC filter cube and images were taken at 200× magnification. Slides were blinded prior to imaging, and lung tissue areas of similar densities without large calibur vessels or bronchi were selected for quantification. To remove the autofluorescent background, images were captured using DAPI and FITC filter cubes at 200× magnification. ImageJ Image Calculator was utilized to subtract background autofluorescence from collagen fibers. After the background was removed, images were converted to 8-bit. Collagen fiber length, width, and number were measured using CT-FIRE detection software (LOCI; Madison, WI, USA) [35].

### 2.11. Statistical Analysis

Results were expressed as mean ± SEM unless stated. Data were tested with the Kolmogorov–Smirnov test for normality. Unless stated in the figure legends, statistical differences were determined using Student’s *t*-test for comparison of two groups or Analysis of Variance (ANOVA) with Tukey’s multiple comparison post-test for multiple groups. Differences in tumor growth rates and body weight differences over time were detected using two-way ANOVA with Tukey’s post hoc test. For serum treatments, differences were detected using paired t-tests. For flow cytometry experiments, outliers were detected using Grubb’s test for outliers. Sample numbers (n) are included in the figure legends for each experiment. *p*-values of 0.05 or less were considered significant. Statistical analyses were conducted using GraphPad Prism 8.3.1 (GraphPad Software, RRID:SCR_002798).

## 3. Results

### 3.1. Obesity Increases Mammary Tumor Metastasis 

**Figure 1:**
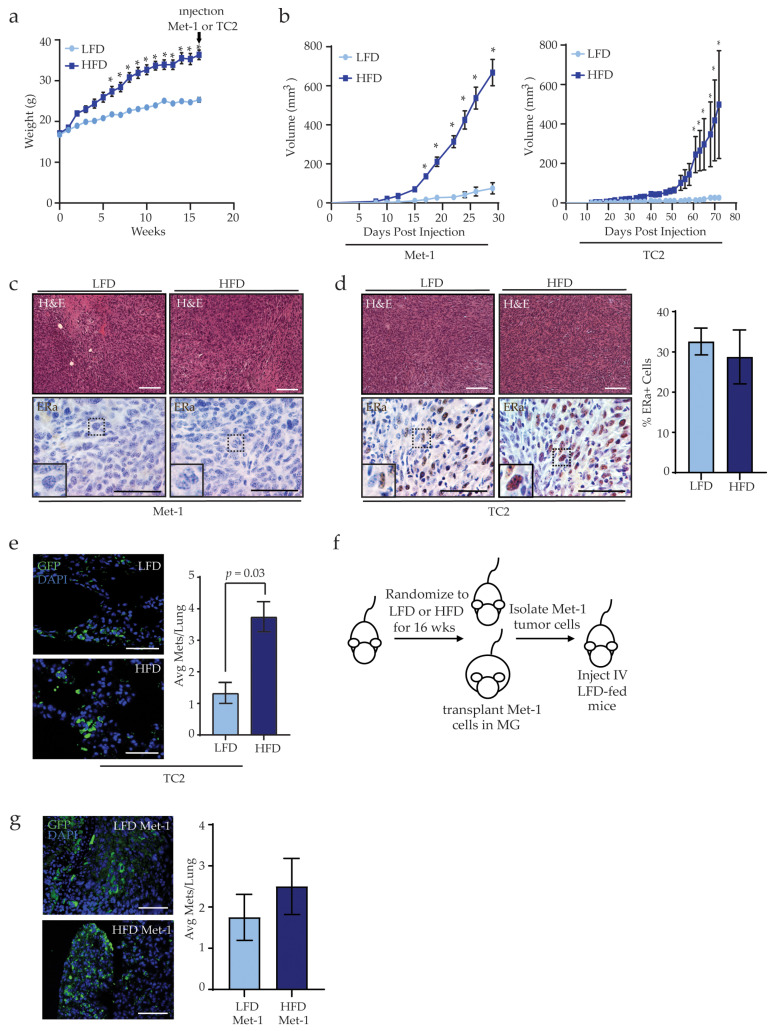
Obesity increases breast cancer metastasis. (**a**) Weight gain of female mice fed either the low-fat diet (LFD) or high-fat diet (HFD) for 16 weeks prior to tumor cell transplantation (arrow; n = 8 mice/group, * *p* < 0.05). (**b**) Growth curves of Met-1 or TC2 tumor cells transplanted into mammary glands of LFD- or HFD-fed female mice (n = 4 mice/group, * *p* < 0.05). (**c**) Representative images of Met-1 tumors stained with hematoxylin and eosin (H&E) or immunohistochemistry (IHC) to detect estrogen receptor alpha (ERα). (**d**) Representative images of TC2 tumors stained with H&E or IHC for ERα. Quantification of ERα^+^ cells in tumors from LFD- or HFD-fed mice (n = 4 mice/group). (**e**) Quantification of metastatic foci of green fluorescent protein (GFP)-expressing TC2 tumor cells in lungs of LFD- and HFD-fed mice (n = 4 mice/group). (**f**) Schematic of experiment to inject tumor cells into the tail vein of LFD-fed mice. (**g**) Quantification of Met-1 metastatic foci in lungs of LFD-fed mice (n = 5 mice/group). Magnification bars = 50 μm.

To examine how obesity impacts mammary tumor growth and metastasis, we utilized a high-fat diet (HFD) model of obesity and implanted mammary tumor cell lines into the inguinal mammary glands of mice. Three-week-old female FVB/N mice were fed either a low-fat diet (LFD) or HFD for 16 weeks to induce obesity. HFD-fed mice gained significantly more weight than LFD-fed mice at 6 weeks after starting the HFD (Figure 1a). We have previously shown that after 16 weeks, HFD-fed female FVB/N mice have increased mammary gland weights, larger adipocyte diameters, and elevated numbers of crown-like structures compared to LFD-fed mice [36]. After 16 weeks, mice were randomized to receive transplants of either Met-1 or TC2 tumor cells into their mammary fat pads, then the mice continued on their respective diets (Figure S1a,b). Consistent with our previous study [31], Met-1 and TC2 mammary tumors from HFD-fed mice grew significantly faster than tumors from LFD-fed mice (Figure 1b), indicating that obesity promotes tumor growth. 

Following transplantation, no significant differences were observed histologically among Met-1 or TC2 tumors from LFD- and HFD-fed mice. Met-1 tumor cells were derived from a MMTV-PyMT tumor and did not express estrogen receptor alpha (ERα) [30]. Consistent with this previous study, we did not observe ERα expression within tumors of LFD- or HFD-fed mice (Figure 1c). In contrast, TC2 tumor cells express ERα in culture [32] and in tumors from LFD- and HFD-fed mice (Figure 1d). No differences were observed in the percentage of ERα-expressing cells in TC2 tumors from LFD- or HFD-fed mice (Figure 1d). These data indicate that obesity enhances the growth of both ERα^+^ and ERα^−^ tumors. 

Clinical evidence suggests that obesity increases the incidence of metastatic breast cancer [5,6]. We have previously shown that HFD-fed mice orthotopically transplanted with Met-1 tumor cells develop significantly more lung metastases than LFD-fed mice [31]. Similarly, HFD-fed mice had significantly more TC2 metastatic foci than LFD-fed mice (*p* = 0.03, Figure 1e). The metastases were variable in size, and diet did not significantly affect the sizes of metastatic foci. These results indicate that obesity also promotes pulmonary metastasis, in addition to accelerating mammary tumor growth. 

Since obesity has been associated with promoting metastasis-initiating cells in breast cancer [31,37], we examined the ability of tumor cells isolated from end-stage tumors from LFD- and HFD-fed mice to establish metastases. Met-1 tumor cells were isolated from the primary tumors of mice fed either LFD or HFD and dissociated primary tumor cells were injected into the tail veins of recipient mice fed an LFD (Figure 1f). After 8 weeks, the lungs of transplanted mice exhibited no significant differences in the average number of metastatic foci, irrespective of the source of the tumor cells (Figure 1g). These data suggest that tumor extrinsic factors may contribute to metastasis under conditions of obesity. 

### 3.2. Obesity Enhances Myeloid Lineage Cells during Metastasis 

**Figure 2:**
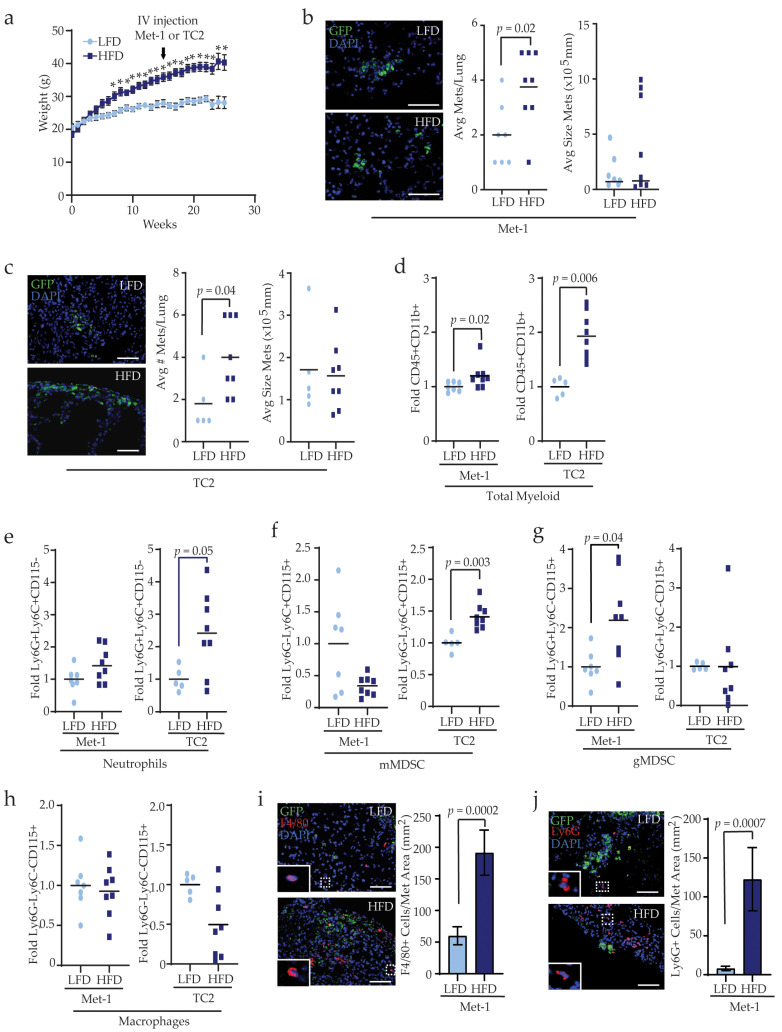
Obesity enhances myeloid lineage cells during metastasis. (**a**) Weight gain of mice fed LFD (n = 12 mice) or HFD (n = 16 mice). Arrow indicates the time point of tail vein injections of Met-1 or TC2 cells (* *p* < 0.05). Quantification of number and size of GFP^+^ metastatic foci in lungs of LFD- and HFD-fed mice injected with Met-1 (**b**) n = 7 mice LFD; n = 8 mice HFD) or TC2 (**c**) n = 5 mice LFD; n = 8 mice HFD) tumor cells. Flow cytometry quantification of CD45^+^CD11b^+^ myeloid lineage cells (**d**) Ly6G^+^Ly6C^+^CD115^−^ neutrophils (**e**), Ly6G^−^Ly6C^+^CD115^+^ monocytic myeloid-derived suppressor cells (monocytic myeloid-derived suppressor cells (mMDSCs), (**f**) Ly6G^+^Ly6C^−^CD115^+^ granulocytic MDSC (granulocytic MDSCs (gMDSCs), and (**g**) Ly6G^−^Ly6C^−^CD115^+^ macrophages (**h**) in lungs from mice injected with either Met-1 or TC2 tumor cells. Results were normalized to CD45^+^CD11b^+^ cells and expressed as fold changes compared to controls. (**i**) Quantification of F4/80^+^ macrophages surrounding lung metastases (n = 5 mice/group). (**j**) Quantification of Ly6G^+^ neutrophils and gMDSC surrounding lung metastases. Magnification bars = 50 μm.

To assess how obesity impacts the lungs to facilitate metastatic colonization, 3-week-old female FVB/N mice were fed a LFD or HFD for 16 weeks and then Met-1 or TC2 tumor cells were injected into the tail vein to generate lung metastasis in the absence of a primary tumor. Mice continued on their respective diets following injection of tumor cells (Figure 2a). Metastases were given time to establish, then lung tissue was collected, and metastases were quantified in tissue sections. HFD-fed mice had significantly increased numbers of lung metastases of variable size than LFD-fed mice after injections of either Met-1 (*p* = 0.02, Figure 2b) or TC2 tumor cell lines (*p* = 0.04, Figure 2c). These results suggest that even in the absence of a primary tumor, obesity promotes lung metastatic colonization. 

Myeloid lineage cells help to promote tumor cell survival and growth at metastatic sites [20]. To determine the impact of obesity on myeloid lineage cells in pulmonary metastases, lungs from mice injected with tumor cells were dissociated into single cells, stained to detect CD45, CD11b, Ly6G, Ly6C, and CD115, and myeloid lineage cell populations and GFP^+^ metastatic cells were analyzed using flow cytometry (Figure S2). 

Consistent with measurements of metastatic colonies, GFP^+^ metastatic cells were variable in number in lungs of LFD- and HFD-fed mice of both models (Figure S2b,c). In HFD-fed mice injected with either Met-1 or TC2 tumor cells, the total CD45^+^CD11b^+^ myeloid lineage cell population was significantly increased compared to LFD-fed mice (Figure 2d), indicating that obesity enhances myeloid cell recruitment during metastatic outgrowth. However, changes in specific myeloid lineage subpopulations differed with respect to the parental tumor cell line. While no significant difference in neutrophils were observed between LFD- and HFD-fed mice bearing Met-1 metastases, HFD-fed mice with TC2 metastases had significantly increased Ly6G^+^Ly6C^+^CD115^−^ neutrophils compared to LFD-fed mice (*p* = 0.05, Figure 2e). Similarly, the population of Ly6G^−^Ly6C^+^CD115^+^ mMDSCs was comparable between HFD- and LFD-fed Met-1 metastases-bearing mice, while mMDSCs were significantly elevated in HFD-fed mice with TC2 metastases compared to LFD-fed mice (*p* = 0.003, Figure 2f). Further, the population of Ly6G^+^Ly6C^−^CD115^+^ gMDSCs was significantly increased in HFD-fed mice with Met-1 metastases compared to those from LFD-fed mice (*p* = 0.04, Figure 2g); however, there was no observed difference in gMDSCs in lungs from TC2 injected HFD- or LFD-fed mice (Figure 2g). In contrast, Ly6G^−^Ly6C^−^CD115^+^ macrophages were unaltered between LFD- and HFD-fed mice injected with either Met-1 or TC2 tumor cells (Figure 2h). Together, these data suggest that while obesity promotes myeloid lineage cell recruitment into lung tissue during metastasis, enrichment for specific immune cell types may depend upon properties of the tumor cells within the metastases. 

To assess spatial relationships between myeloid lineage cells and pulmonary metastases, we examined populations of myeloid lineage cells using immunofluorescence. Since CD115 is expressed on numerous cell types including macrophages, mMDSCs, and gMDSCs [38], we utilized F4/80 expression to detect macrophages, which is expressed on both interstitial and alveolar lung macrophages [39]. Tissue surrounding Met-1 and TC2 metastases in lungs of HFD-fed mice demonstrated significantly greater F4/80^+^ macrophage recruitment than metastases from LFD-fed mice (Figure 2i and Figure S3a). Further, Ly6G^+^ cells, including both gMDSCs and neutrophils, were increased around metastases from HFD-fed mice compared to metastases from LFD-fed mice (Figure 2j and Figure S3b). These data indicate that recruitment of myeloid lineage cells, including gMDSC and mMDSC, to metastatic sites in the lungs is enhanced by obesity.

### 3.3. Obesity Alters the Complementarity of Myeloid Lineage Cells in the Lungs Prior to Metastasis

**Figure 3:**
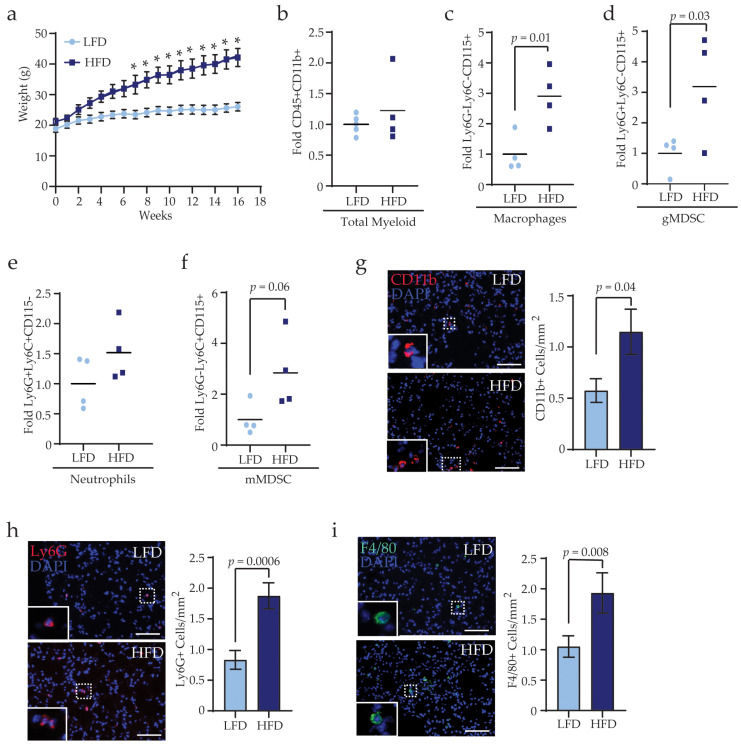
Obesity alters the compliment of myeloid lineage cells in lungs prior to metastasis. (**a**) Weight gain of female mice fed either LFD or HFD for 16 weeks (n = 8 mice/group, * *p* < 0.05). Flow cytometry quantification of CD45^+^CD11b^+^ myeloid lineage cells (**b**), Ly6G^−^Ly6C^−^CD115^+^ macrophages (**c**), Ly6G^+^Ly6C^−^CD115^+^ gMDSC (**d**), Ly6G^+^Ly6C^+^CD115^−^ neutrophils (**e**), and Ly6G^−^Ly6C^+^CD115^+^ mMDSC (**f**). Results were normalized to CD45^+^CD11b^+^ cells and expressed as fold change compared to controls (n = 4 mice/group). Quantification of CD11b^+^ myeloid lineage cells (**g**), Ly6G^+^ neutrophils and gMDSCs (**h**), and F4/80^+^ macrophages (**i**) in lungs of LFD- and HFD-fed mice (n = 5 mice/group). Magnification bars = 50 μm.

In the bone marrow, obesity enhances myeloid progenitor cell proliferation and upregulates cytokine production [40,41], suggesting that immune cells are systemically increased as a result of obesity. We hypothesized that obesity may promote trafficking of myeloid lineage cells into the lungs prior to the onset of primary tumors, which may enhance metastasis. To examine myeloid lineage cell recruitment into lungs prior to metastasis formation, we collected lungs from LFD- and HFD-fed tumor-naïve mice (Figure 3a) and performed flow cytometry to quantify myeloid lineage cell populations. In contrast to metastatic lungs, there was no significant difference in total CD45^+^CD11b^+^ myeloid lineage cells among lungs from LFD- and HFD-fed mice (Figure 3b). However, Ly6G^−^Ly6C^−^CD115^+^ macrophages (*p* = 0.01, Figure 3c) and Ly6G^+^Ly6C^−^CD115^+^ gMDSCs (*p* = 0.03, Figure 3d) were significantly increased while Ly6G^+^Ly6C^+^CD115^−^ neutrophils and Ly6G^−^Ly6C^+^CD115^+^ mMDSCs were not significantly different in lungs of HFD-fed mice compared to those from LFD-fed mice (Figure 3e,f). These data indicate that obesity promotes the recruitment of macrophages and gMDSCs to the lungs prior to metastasis formation.

To assess the localization of myeloid lineage cells in tumor-naïve mice, we examined immune cell markers in lungs sections from LFD- and HFD-fed mice using immunofluorescence. HFD-fed mice demonstrated significantly increased numbers of CD11b^+^ myeloid lineage cells per area of lung tissue compared to LFD-fed mice (*p* = 0.03, Figure 3g). Further, recruitment of Ly6G^+^ gMDSCs and neutrophils (*p* = 0.0006, Figure 3h) and F4/80^+^ macrophages (*p* = 0.008, Figure 3i) were significantly increased in lung tissue of HFD-fed mice compared to LFD-fed mice. These data suggest that obesity alters myeloid lineage cell trafficking into the lungs before tumor formation. 

### 3.4. Obesity Activates Stromal Cells within the Lungs through TGFβ1 Expression

**Figure 4:**
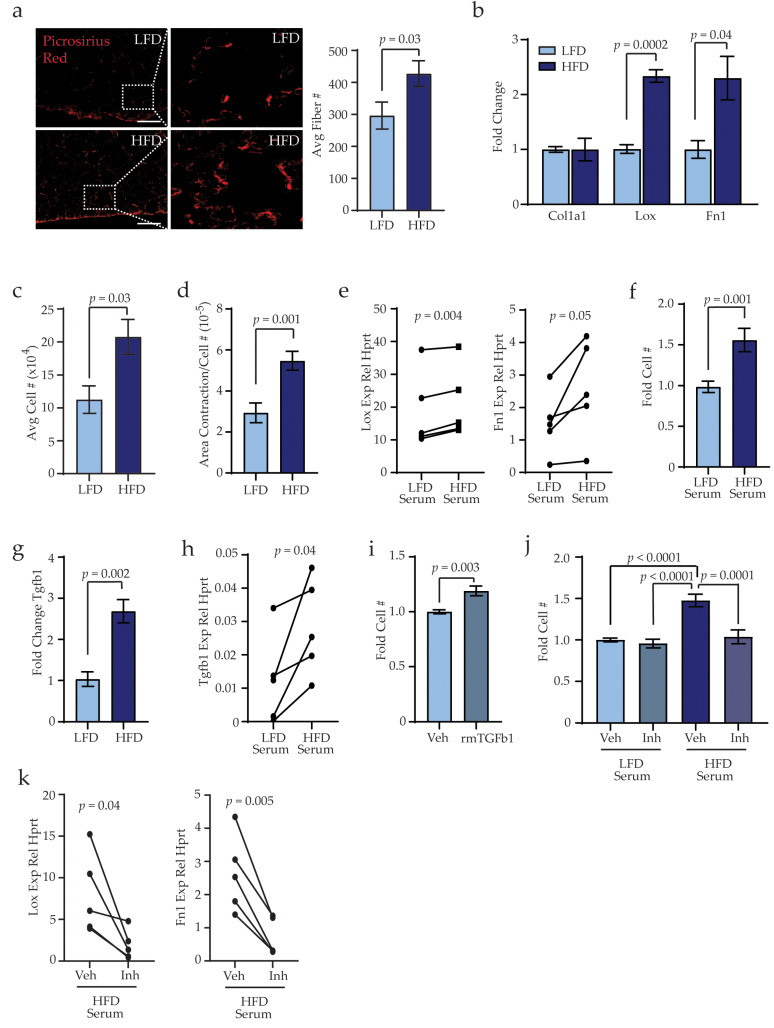
Obesity alters lung stromal cells within the lungs in the absence of tumor formation. (**a**) Quantification of picrosirius red-stained collagen fibers in lung sections of LFD- and HFD-fed non-tumor-bearing mice (n = 5 mice/group). (**b**) Expression of extracellular matrix (ECM) components from primary lung stromal cells. Expression differences were normalized to hypoxanthine-guanine phosphoribosyltransferase (*Hprt*) and represented as fold change from controls (n = 6 mice/group). (**c**) Cell numbers of isolated lung stromal cells grown in culture for 6 days (n = 3 mice/group). (**d**) Average area of collagen contracted from lung stromal cells (n = 3 mice/group). (**e**) *Lox* and *Fn1* expression in lung stromal cells isolated from LFD-fed mice treated with serum from LFD- or HFD-fed mice. Represented as relative expression compared to *Hprt* (n = 5 mice/group; paired t-test). (**f**) Cell numbers of isolated lung stromal cells from LFD-fed mice treated with serum from LFD- or HFD-fed mice for 6 days (n = 5 mice/group). (**g**) Expression of transforming growth factor beta-1 (*Tgfβ1*) from primary lung stromal cells. Expression differences were normalized to *Hprt* and represented as fold change from controls (n = 6 mice/group). (**h**) *Tgfβ1* expression in lung stromal cells isolated from LFD-fed mice treated with serum from LFD- or HFD-fed mice. Represented as relative expression compared to *Hprt* (n = 5 mice/group; paired t-test). (**i**) Lung stromal cells from LFD-fed mice treated with vehicle (veh) or recombinant mouse (rm) TGFβ1 (n = 3 mice/group). (**j**) Lung stromal cells from LFD-fed mice treated with serum from LFD- or HFD-fed mice supplemented with veh or TGFβ inhibitor (inh) SB431542 (n = 3 mice/group). (**k**) *Lox* and *Fn1* expression in lung stromal cells isolated from LFD-fed mice treated with serum from LFD- or HFD-fed mice. Represented as relative expression compared to *Hprt* (n = 5 mice/group; paired t-test). Magnification bars = 50 μm.

In tumor-bearing mice, premetastatic niches in the lungs have been shown to promote recruitment of bone marrow-derived immune cells through increased collagen deposition and fibrosis [42]. To determine how obesity impacts collagen deposition within the lung microenvironment, we stained lung tissue sections of tumor-naïve LFD- and HFD-fed mice using picrosirius red. The number of collagen fibers within lung tissue was significantly increased in HFD-fed mice compared to lungs from LFD-fed mice (*p* = 0.03, Figure 4a). Collagen fiber length and width remained unchanged between lungs of LFD- and HFD-fed mice (Figure S4a). These results suggest that obesity increases accumulation of collagen fibers within lung tissue of tumor-naïve mice. 

To examine how obesity may impact lung stromal cells, stromal cells were isolated from lung tissue of LFD- and HFD-fed mice and cultured to generate adherent cells. These adherent lung stromal cell cultures did not contain detectable transcripts for *Cnn1*, *Cd31*, and *Epcam*, and only low *Cd45* expression compared to splenic tissue (Figure S4b), indicating that short-term culture of lung stromal cells depletes epithelial, endothelial, pericyte, and immune cell populations. Although lungs of HFD-fed mice exhibited higher numbers of collagen fibers, lung stromal cells from HFD-fed mice expressed similar levels of *Col1a1* compared to those from LFD-fed mice (Figure 4b). However, expression of *Lox* (lysyl oxidase), an enzyme that increases ECM crosslinking and collagen stability, was significantly increased in lung stromal cells from HFD-fed mice compared to LFD-fed mice (*p* = 0.0002, Figure 4b). Additionally, lung stromal cells from HFD-fed mice demonstrated significantly increased expression of *Fn1* (fibronectin; *p* = 0.04, Figure 4b), which has been implicated as an ECM component of cancer-induced premetastatic niches [43]. 

In culture, lung stromal cells from HFD-fed mice demonstrated significantly increased cell numbers after 6 days compared to those from LFD-fed mice, suggesting increased proliferation of stromal cells from HFD-fed mice (*p* = 0.03, Figure 4c). To test how obesity impacted lung stromal cell function, lung stromal cells were plated into collagen gels and contractility of the gel was measured after 7 days. Lung stromal cells from HFD-fed mice demonstrated significantly increased contraction of collagen gels compared to lung stromal cells from LFD-fed mice (*p* = 0.001, Figure 4d). Together, these results indicate that obesity alters ECM deposition and function of lung stromal cells.

In obesity, adipose tissue is chronically inflamed, and multiple inflammatory cytokines and growth factors are elevated systemically in serum [44,45]. We hypothesized that circulating inflammatory factors may promote the changes we observed in lung stromal cells from HFD-fed mice. To test this hypothesis, we cultured lungs stromal cells from LFD-fed mice and treated them with serum isolated from either LFD- or HFD-fed mice. Consistent with our in vitro analyses of lung stromal cells isolated from HFD-fed mice, lung stromal cells from LFD-fed mice that were treated with serum from HFD-fed mice demonstrated significantly increased expression of *Lox* (*p* = 0.004) and *Fn1* (*p* = 0.05) compared to the same cells treated with serum from LFD-fed mice (Figure 4e). Lung stromal cells from LFD-fed mice treated with serum from HFD-fed mice also had increased cell numbers after 6 days, compared to the paired lung stromal cells treated with serum from LFD-fed mice (*p* = 0.001; Figure 4f), suggesting that exposure to serum from HFD-fed mice enhanced lung stromal cell proliferation rates. Together, these results suggest that exposure to circulating inflammatory cytokines and/or growth factors from obese mice promotes expression of ECM remodeling components as well as functional changes of lung stromal cells. 

Transforming growth factor beta (TGFβ) has been implicated in increased ECM production in pathological conditions of lung fibrosis, with TGFβ1 as the predominant TGFβ isoform expressed [46]. Lung stromal cells from HFD-fed mice expressed significantly higher levels of *Tgfβ1* compared to those from LFD-fed mice (*p* = 0.002, Figure 4g). While TGFβ1 levels were similar in sera from LFD- and HFD-fed mice (Figure S4c), treatment of lung stromal cells from LFD-fed mice with serum from HFD-fed mice resulted in significantly increased *Tgfβ1* expression (*p* = 0.04, Figure 4h). Functionally, culture of lung stromal cells from LFD-fed mice with recombinant mouse TGFβ1 significantly enhanced cell numbers after 6 days (*p* = 0.003, Figure 4i). Further, treatment of lung stromal cells with serum from HFD-fed mice in the presence of the TGFβ inhibitor SB431542 resulted in significantly reduced cell numbers compared to serum from HFD-fed mice with vehicle (*p* = 0.0001, Figure 4j). In contrast, no differences were observed in cell numbers of lung stromal cells from LFD-fed mice treated with serum from LFD-fed mice supplemented with vehicle or TGFβ inhibitor (Figure 4j). In addition, treatment of lung stromal cells with se-rum from HFD-fed mice in the presence of SB431542 resulted in significantly reduced expression of *Lox* and *Fn1* compared to those treated with vehicle (Figure 4k). Together, these results suggest that inflammatory mediators from obese mice increase TGFβ1 expression within lung stromal cells to promote proliferation and collagen and ECM deposition. 

### 3.5. Obesity-Activated Lung Stromal Cells Enhance Migration of Bone Marrow and Tumor Cells

**Figure 5:**
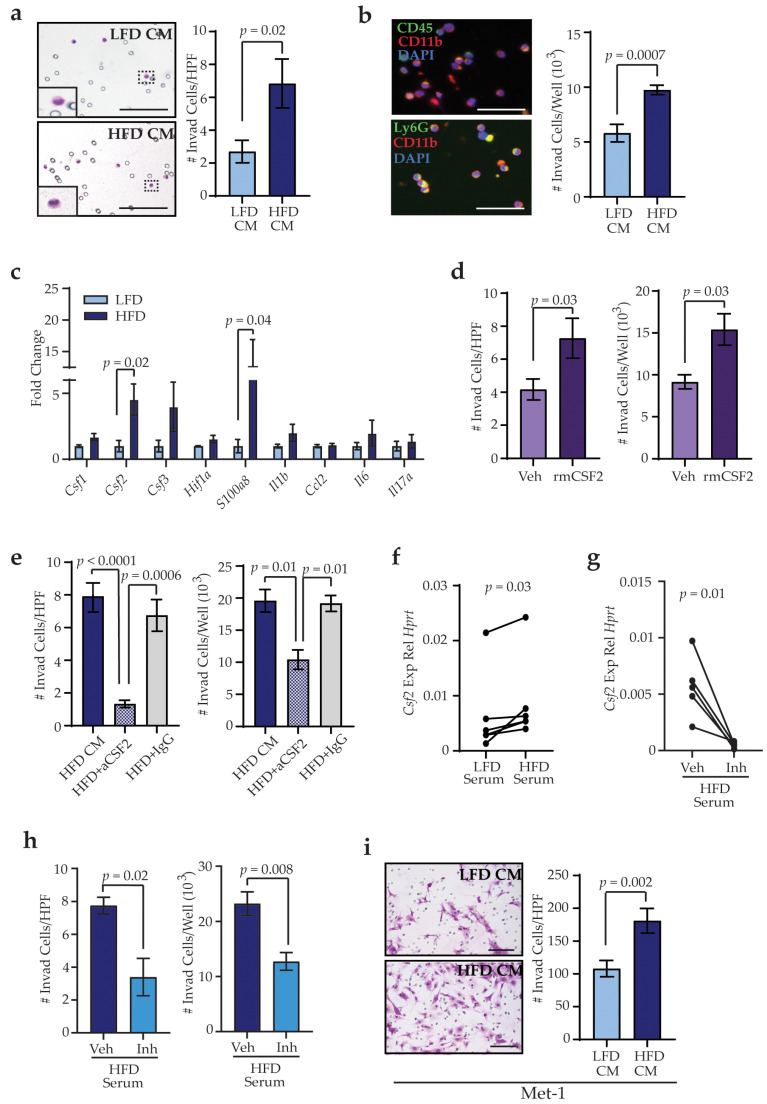
Lung stromal cells from HFD-fed mice recruit myeloid lineage cells through colony-stimulating factor 2 (CSF2) expression. (**a**) Quantification of adherent bone marrow-derived cells invading toward conditioned media from lung stromal cells from LFD- and HFD-fed mice in each high-power field (HPF, n = 5 mice/group). (**b**) Quantification of nonadherent bone marrow-derived cells in conditioned media from lung stromal cells (n = 5 mice/group). (**c**) Expression levels of cytokines from primary lung stromal cells. Differences were normalized to *Hprt* and represented as fold change from controls (n = 6–7 mice/group). (**d**) Quantification of adherent and nonadherent bone marrow-derived cells invading toward vehicle (veh) or recombinant mouse (rm) CSF2 (n = 3 experiments). (**e**) Quantification of adherent and nonadherent bone marrow-derived cells invading in response to conditioned media isolated from lung stromal cells from HFD-fed mice supplemented with vehicle, IgG antibodies, or blocking antibodies for CSF2 (n = 3 experiments). (**f**) *Csf2* expression in lung stromal cells isolated from LFD-fed mice treated with serum from LFD- or HFD-fed mice. Represented as relative expression compared to *Hprt* (n = 5 mice/group; paired t-test). (**g**) Csf2 expression in lung stromal cells treated with serum from HFD-fed mice supplemented with vehicle or TGFβ inhibitor (inh) SB431542. Represented as relative expression compared to *Hprt* (n = 5 mice/group; paired t-test). (**h**) Quantification of adherent and nonadherent bone marrow-derived cells invading in response to conditioned media isolated from lung stromal cells treated with serum from HFD-fed mice supplemented with vehicle or SB431542 (n = 3 experiments). (**i**) Quantification of Met-1 cells invading toward conditioned media from lung stromal cells (n = 5 mice/group). Magnification bars = 50 μm.

To assess how obesity-induced changes in lung stromal cells may aid in trafficking of bone marrow cells into the lungs, we tested the ability of bone marrow cells isolated from LFD-fed mice to migrate toward secreted factors from lung stromal cells of LFD- and HFD-fed mice through collagen-coated transwells. We collected conditioned media from lung stromal cells, and we examined the ability of isolated bone marrow cells to migrate in response to conditioned media. Immune cells adherent to the bottom surface of the membranes were significantly increased in response to conditioned media from lung stromal cells from HFD-fed mice compared to LFD-fed mice (*p* = 0.02, Figure 5a). We also observed CD45^+^ bone marrow cells that invaded through the collagen into the conditioned media (Figure 5b). Approximately 90% of these cells expressed marker CD11b, consistent with cells of the myeloid lineage, and about 75% of the cells also expressed Ly6G (Figure 5b). Although conditioned media from HFD-fed mice did not significantly alter the types of cells that invaded through the collagen, the number of cells that were present in the conditioned media of lung stromal cells from HFD-fed mice was significantly increased compared to controls (*p* = 0.0007, Figure 5b). These data suggest that obesity-altered lung stromal cells enhance trafficking of bone marrow-derived immune cells into the lungs. 

Stromal cells in premetastatic secondary organs exhibit altered production and secretion of cytokines in response to tumor-derived factors [12], as metastatic tumor cells are reliant on local stromal cells to successfully colonize the organ [47]. Lung stromal cells from HFD-fed mice demonstrated significantly increased expression of *Csf2* (*p* = 0.02), which is elevated in local sites of inflammation and is implicated in myeloid lineage cell recruitment and maturation [48], and *S100a8* (*p* = 0.04), which modulates inflammatory responses through immune cell recruitment into tissues [49] (Figure 5c). Media containing recombinant mouse CSF2 significantly increased invasion of immune cells through collagen-coated transwells as either adherent cells (*p* = 0.03) or into the media (*p* = 0.03, Figure 5d). In addition, supplementation of conditioned media from lung stromal cells of HFD-fed mice with blocking antibodies for CSF2 significantly reduced invasion through transwells both for adherent cells (*p* = 0.0006) as well as cells within the media containing the blocking antibodies (*p* = 0.01) as compared to conditioned media from lung stromal cells of HFD-fed mice treated with IgG control antibodies (Figure 5e). Together, these results suggest that lung stromal cells from obese mice enhance myeloid lineage cell recruitment through elevated expression of CSF2.

Since lung stromal cells from LFD-fed mice were functionally altered by exposure to serum from HFD-fed mice, we hypothesized that serum collected from HFD-fed mice may induce *Csf2* expression in lung stromal cells from LFD-fed mice. As shown in Figure 5f, lung stromal cells from LFD-fed mice significantly increased expression of *Csf2* in response to treatment with serum from HFD-fed mice, compared to serum from LFD-fed mice (*p* = 0.03). Together, these results suggest that circulating factors induced by obesity can promote *Csf2* expression in lung stromal cells. 

As TGFβ1 has been shown to enhance expression of *Csf2* in other cell types [50,51], we hypothesized that reduced TGFβ1 activity would decrease *Csf2* expression. To examine this question, lung stromal cells were treated with serum from HFD-fed mice supplemented with either vehicle or SB431542. Compared with vehicle-treated cells, lung stromal cells treated with SB431542 demonstrated significantly decreased expression of *Csf2* (*p* = 0.01, Figure 5g). Consistent with decreased expression of *Csf2*, migration of bone marrow-derived cells through transwells was significantly reduced in response to conditioned media collected from lung stromal cells treated with SB431542 in the presence of serum from HFD-fed mice (Figure 5h). These results suggest that CSF2-dependent recruitment by lung stromal cells is enhanced through elevated TGFβ1 activity.

During lung metastasis, disseminated tumor cells leave blood vessels and invade into the lung stroma. We hypothesized that activated lung stromal cells from obese mice may also promote the invasion of tumor cells. Similar to our observations of immune cells, increased numbers of Met-1 (*p* = 0.002; Figure 5i) and TC2 (*p* = 0.005; Figure S4d) tumor cells invaded through collagen-coated transwells in response to factors secreted by lung stromal cells from HFD-fed mice. These results suggest that obesity enhances recruitment of immune cells and tumor cells together into the lung stroma through activation of lung stromal cells. 

## 4. Discussion

Prior to metastasis, primary tumors create niches in distal organs conducive to metastatic colonization. In the absence of a primary mammary tumor, we observed that obesity altered the microenvironment of the lungs with similarities to tumor-induced premetastatic niches. Within lung tissue, obesity enhanced recruitment of myeloid lineage cells, in particular macrophages and gMDSCs, as well as increased deposition of collagen fibers. Lung stromal cells isolated from obese mice demonstrated elevated expression of *Lox* and *Fn1*, similar to lung fibroblasts within premetastatic niches [52]. These changes in the immune microenvironment and ECM under conditions of obesity enhanced the ability of ERα^+^ and ERα^−^ tumor cells to establish metastatic colonization. Within the mammary gland, obesity also promoted the rapid growth of ERα^+^ and ERα^−^ primary mammary tumors, and obesity has been shown to enhance cancer stem-like cells within mammary tumors [31,53]. Since cancer stem-like cells may increase metastatic potential [54], these results suggest that obesity enhances lung metastasis both though promotion tumor cells with increased metastatic potential as well as by establishment of favorable conditions for metastasis. These obesity-induced conditions may contribute to the clinically observed increased risk for metastasis in obese breast cancer patients [5,6].

Similar to cancer, obesity induces nonresolving inflammation, resulting in enhanced circulating numbers of bone marrow-derived myeloid lineage cells [40,41]. Our results indicated that these bone marrow-derived cells may be recruited from the circulation into lung tissue by lung stromal cells in obesity through locally elevated CSF2 as well as S100A8, which has been shown to promote accumulation of myeloid lineage cells at metastatic sites in vivo [55,56]. Recent studies have identified that lung tissue expresses higher expression levels of *Csf2* under conditions of obesity, potentially through elevated recruitment of immune cell populations that also express *Csf2* [44]. Our studies extend these findings to suggest that lung fibroblasts within the stroma promote the recruitment of the immune cells into the tissue. In premetastatic niches, recruited myeloid lineage cells prepare distant organs for metastatic seeding [12,20]. Macrophages are necessary for metastatic growth in the lungs [57,58,59], and obesity may alter macrophage function in lung tissue, potentially in response to circulating inflammatory cytokines [60]. In addition, elevated levels of TGFβ1 in lung tissue from lung stromal cells may promote an immunosuppressive phenotype in the recruited macrophages in obesity [61]. gMDSCs have also been shown to enhance metastasis through immunosuppressive effects and vascular remodeling within the lungs [62]. Our studies support a role for increased early recruitment of myeloid lineage cells which may have an immunosuppressive phenotype into lung tissue which may further promote an environment conducive to metastasis under conditions of obesity. 

ECM remodeling in distant organs is essential for metastatic colonization [23]. We observed that obesity enhances collagen deposition and *Lox* expression within lung tissue of tumor-naïve mice. Collagen deposition and stabilization in the lungs through activity of lysyl oxidase has been shown to promote breast cancer cell metastatic colonization [63]. Lung stromal cells from obese tumor-naïve mice also exhibited increased cell numbers with culture and enhanced contractility, which has been observed in lung fibroblasts isolated from lung metastatic sites [52]. Lung stromal cells from obese mice expressed significantly higher levels of *Tgfβ1*, and treatment with serum from obese mice induced *Tgfβ1* expression in lung stromal cells from LFD-fed mice. In models of lung fibrosis, enhanced TGFβ1 expression precedes increased collagen and extracellular matrix deposition [64,65]. These changes in lung fibroblasts from obese mice may be due to exposure to inflammatory cytokines present in serum, as treatment with tumor-derived factors in culture resulting in increased expression of collagen-1A1 and myofibroblast marker smooth muscle actin in lung fibroblasts [22]. We also observed that treatment of lung stromal cells with a TGFβ1 inhibitor significantly reduced expression of *Lox* and *Fn1*. Leptin, which is an adipokine that is increased in the serum of obese individuals [66], may also play a role [67]. In pulmonary fibrosis, lung fibroblasts are activated through increases in microRNAs [68,69], immune cells [25], and serum cytokines [26], and further studies are needed to determine the mediators of obesity-induced lung stromal activation. Interestingly, although we observed significantly increased incidence of lung metastasis under conditions of obesity, we did not observe an enhanced metastatic size. In the mammary gland, the primary mammary tumors grew significantly faster in HFD-fed mice compared to LFD-fed mice. It is possible that elevated adipokines within the microenvironment of the obese mammary gland play a critical role in tumor cell growth. Further comparison of the lung and mammary microenvironments may provide insight into these differences in tumor cell growth.

Metastasis is the primary cause of breast cancer mortality and identifying points of intervention to reduce metastatic risk in obese breast cancer patients is critical. Given that obesity is a chronic inflammatory disease, targeting obesity-induced recruitment of immune cells to the lungs may have therapeutic benefits for obese breast cancer patients. A recent study demonstrated that 10% loss of body mass in a small cohort of morbidly obese individuals resulted in a decrease in systemic inflammatory markers [70], which suggests that weight loss may reduce systemic inflammation. However, other studies have suggested that weight loss may not reverse epigenetic changes induced in obese adipose tissue [71], and the impact of weight loss on the function of lung stromal cells needs to be investigated. Given the similarities that we observed among lung stromal cells in obesity- and cancer-associated fibroblasts, therapeutics in development to target inflammatory characteristics of fibroblasts as well as antifibrotic agents may also have efficacy to limit metastasis in obese breast cancer patients [72,73]. Further studies are necessary to determine how obesity alters stromal cells in the microenvironment of other frequent sites of breast cancer metastases, such as liver and bone. Given that obesity increases the incidence of multiple types of cancer [74], understanding how obesity promotes early metastasis through changes in the metastatic microenvironment may improve treatment options for the rising population of obese cancer patients.

## 5. Conclusions

In non-tumor-bearing mice, lung tissue from obese mice demonstrated increased collagen deposition, which may be due to elevated expression of *Lox* and *Fn1* by lung stromal cells. Obesity-activated lung stromal cells also enhanced recruitment of myeloid-lineage cells through elevated expression of CSF2. Inhibition of TGFβ1 signaling reduced expression of *Csf2* and diminished migration of myeloid-lineage cells in vitro. Together, these studies suggest that obesity promotes an environment in the lungs conducive to metastasis formation, reminiscent of premetastatic niches formed by mammary tumors. Changes in lung stromal cells due to obesity may underlie in part the clinical observation of increased incidence of metastasis in obese breast cancer patients. 

## Data Availability

No new data were created or analyzed in this study. Data sharing is not applicable to this article.

## Supplementary Materials

The following are available online at https://www.mdpi.com/2072-6694/13/5/1005/s1. Figure S1: Weight gain of mice following tumor cell transplants; Figure S2: Flow cytometry gating strategy of non-tumor-bearing and metastatic lungs; Figure S3: Immune cell recruitment surrounding TC2 metastases; Figure S4: Characterization of lung stromal cells from LFD- and HFD-fed mice; Table S1: Primers used for qRT-PCR analyses; Table S2: Antibodies used for flow cytometry, immunohistochemistry and immunofluorescence.
