# Supplementary material for: Obesity-Activated Lung Stromal Cells Promote Myeloid Lineage Cell Accumulation and Breast Cancer Metastasis
Source: Cancers (Basel). 2021 Feb 28;13(5):1005. doi: 10.3390/cancers13051005 (PMC7957630; doi:10.3390/cancers13051005)
Supplement: Supplementary file 1 [file cancers-13-01005-s001.zip › Supplementary Figure 1.pdf]

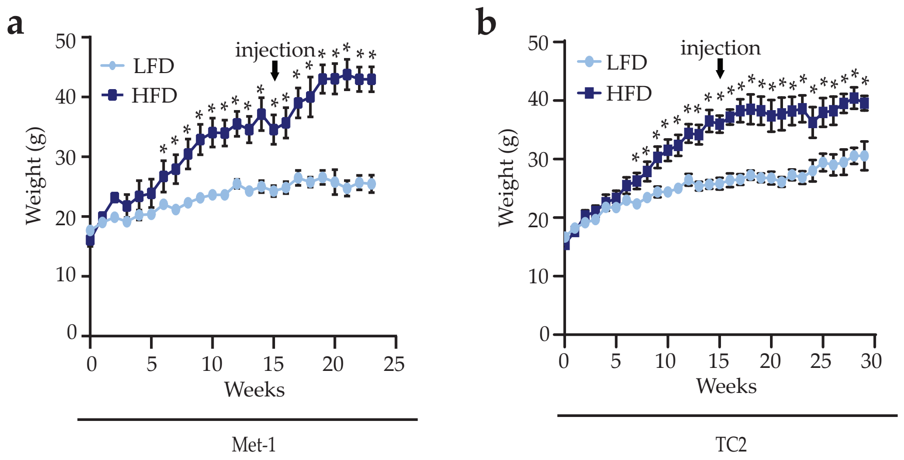

Figure S1. Weight gain of mice following tumor cell transplants. Weight gain of female mice fed either the low-fat (LFD) or high-fat diet (HFD). Mice received tumor cell injections (arrow) of either Met-1 tumor cells (n=4 mice/group) (**a**) or TC2 tumor cells (n=4 mice/group) (**b**) into their inguinal mammary glands after being fed their respective diets for 16 weeks. *$p < 0.05$
