# Supplementary material for: Obesity-Activated Lung Stromal Cells Promote Myeloid Lineage Cell Accumulation and Breast Cancer Metastasis
Source: Cancers (Basel). 2021 Feb 28;13(5):1005. doi: 10.3390/cancers13051005 (PMC7957630; doi:10.3390/cancers13051005)
Supplement: Supplementary file 1 [file cancers-13-01005-s001.zip › Supplementary Figure 2.pdf]

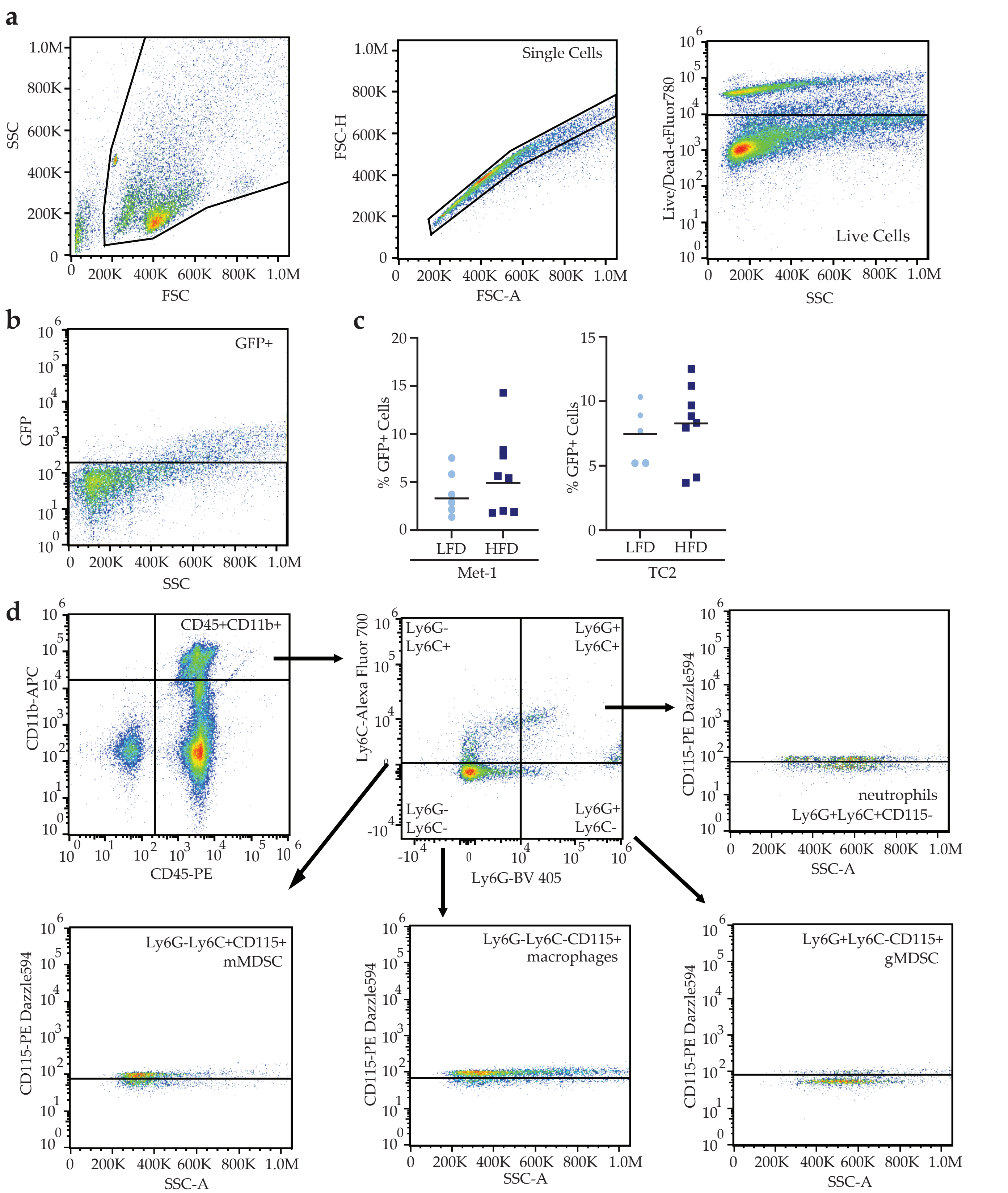

Figure S2. Flow cytometry gating strategy of non-tumor bearing and metastatic lungs. (**a**) Debris was gated out using SSC and FCS. Doublets were gated out using FSC-A and FSC-H, and live cells were negative for viability dye eFluor 780. (**b**) GFP+ cells were identfied in metastatic lungs. (**c**) Percent of GFP+ Live Cells in metastatic lungs. (**d**) PE-conjugated CD45 and APC-conjugated CD11b were plotted to identify CD45+CD11b+ myeloid lineage cells. CD45+CD11b+ cells were gated to determine Ly6G+/-Ly6C+/- populations. Each population was plotted with CD115-PE/Dazzle 594 and SSC-A to identify Ly6G+Ly6C+CD115- neutrophils, Ly6G-Ly6C+CD115+ mMDSC, Ly6G-Ly6C-CD115+ macrophages, and Ly6G+Ly6C-CD115+ gMDSC.
