# Supplementary material for: Obesity-Activated Lung Stromal Cells Promote Myeloid Lineage Cell Accumulation and Breast Cancer Metastasis
Source: Cancers (Basel). 2021 Feb 28;13(5):1005. doi: 10.3390/cancers13051005 (PMC7957630; doi:10.3390/cancers13051005)
Supplement: Supplementary file 1 [file cancers-13-01005-s001.zip › Supplementary Figure 3.pdf]

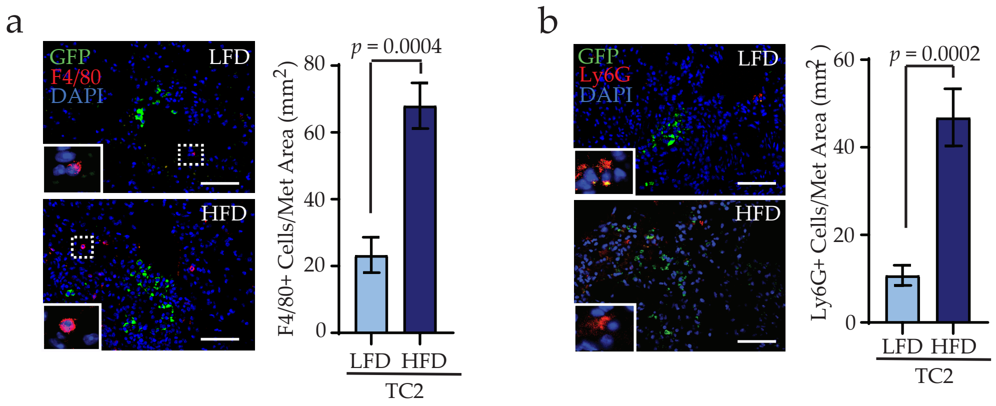

Figure S3. Immune cell recruitment surrounding TC2 tumor cell metastases. (**a**) Quantification of F4/80+ macrophages surrounding lung metastases (n=5 mice/group). (**b**) Quantification of Ly6G+ neutrophils and gMDSC surrounding lung metastases. Magnification bars = 50μm.
