# Supplementary material for: Obesity-Activated Lung Stromal Cells Promote Myeloid Lineage Cell Accumulation and Breast Cancer Metastasis
Source: Cancers (Basel). 2021 Feb 28;13(5):1005. doi: 10.3390/cancers13051005 (PMC7957630; doi:10.3390/cancers13051005)
Supplement: Supplementary file 1 [file cancers-13-01005-s001.zip › Supplementary Figure 4.pdf]

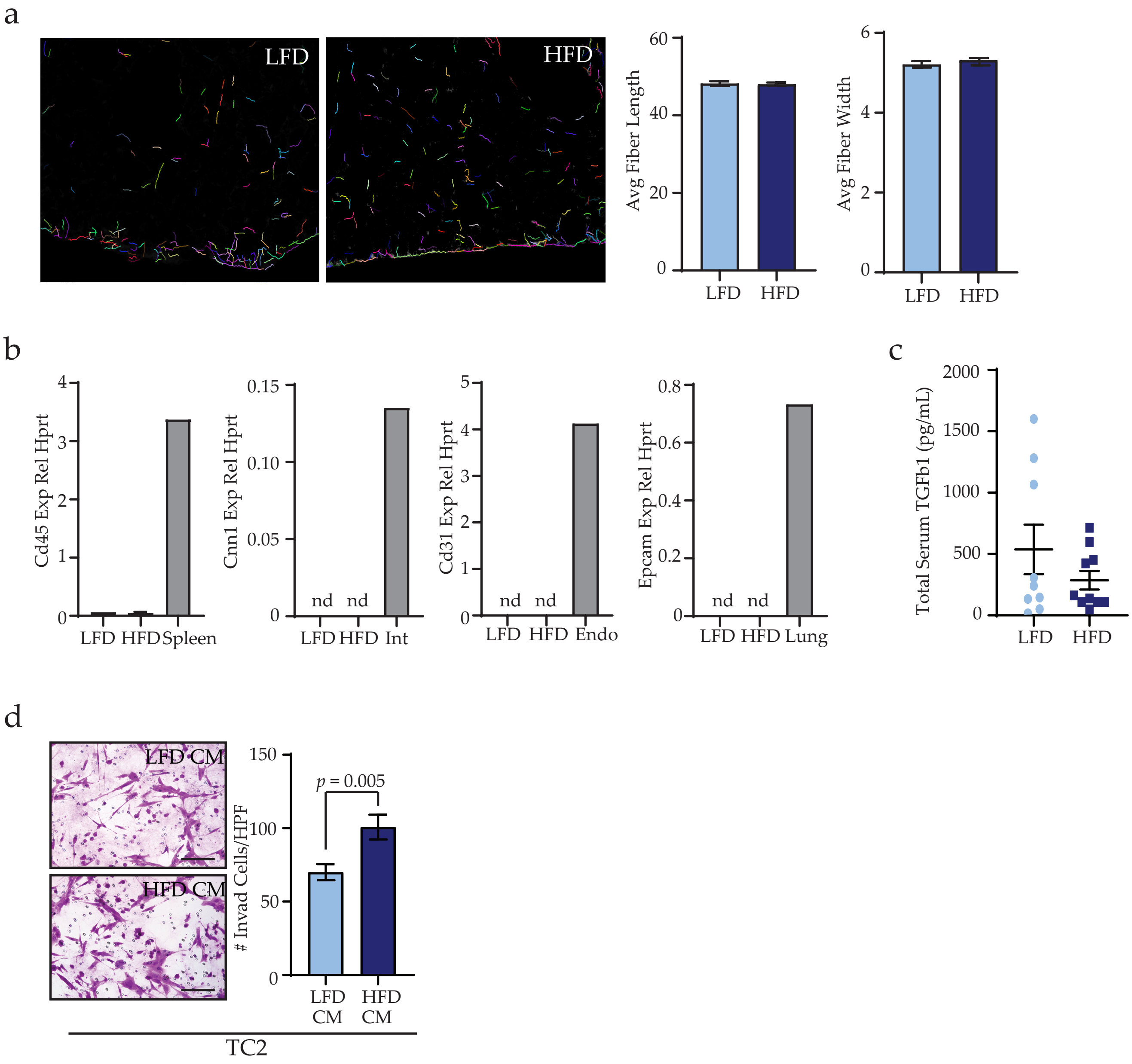

Figure S4. Characterization of lung stromal cells from LFD or HFD-fed mice. (**a**) Quantification of collagen length and width using CT-FIRE in picrosirius red-stained sections of lungs from non-tumor-bearing LFD and HFD-fed mice (n=5 mice/group). (**b**) Expression of markers for immune cells (*Cd45*), endothelial cells (*Cd31*), pericytes (*Cnn1* and *Cd31*), and epithelial cells (*Epcam*) from lung stromal cells isolated from LFD or HFD-fed mice. Positive controls included spleen, intestines (int), primary mouse endothelial cells (endo), and whole lung tissue. Represented as relative expression compared to *Hprt* (n=3 mice/group). (**c**) Concentration of total TGFβ1 protein in serum from LFD and HFD-fed mice (n=9-10 mice/group). (**d**) Quantification of TC2 cells in high-power fields (HPF) invading toward conditioned media from lung stromal cells (n=5 mice/group). Magnification bars = 50µm.
