# Supplementary material for: Obesity-Activated Lung Stromal Cells Promote Myeloid Lineage Cell Accumulation and Breast Cancer Metastasis
Source: Cancers (Basel). 2021 Feb 28;13(5):1005. doi: 10.3390/cancers13051005 (PMC7957630; doi:10.3390/cancers13051005)
Supplement: Supplementary file 1 [file cancers-13-01005-s001.zip › Supplementary Tables.docx]

**Table S1. Primers used for qRT-PCR analyses.**

|  | **Forward 5’-3’** | **Reverse 5’-3’** |
| --- | --- | --- |
| *Hprt* | GAGTCAACGGATTTGGTCGT | GACAAGCTTCCCGTTCTCAG |
| *Csf1* | ACCCAGCTGCCCGTATGAC | TCCTTGGCAATACTCCTGCTC |
| *Csf2* | CAGGGCTGTTTTCCCATCCAT | GCCATGTTCTATCGGGTACTTC |
| *Csf3* | TGTCCTGGCCATTTCGTACC | CAGGTCTAGGCCAAGTGGTG |
| *Hif1a* | AATGCTCAGAGGAAGCGAAAAA | ATCCTTTCACTCGTTTCCAGGAA |
| *S100a8* | TGTCCTCAGTTTGTGCAGAATAAA | TTTATCACCATCGCAAGGAACTC |
| *Il1b* | GCAACTGTTCCTGAACTCAACT | ATCTTTTGGGGTCCGTCAACT |
| *Ccl2* | TTAAAAACCTGGATCGGAACCAA | GCATTAGCTTCAGATTTACGGGT |
| *Il6* | ACAAAGCCAGAGTCCTTCAGAG | GTGAGGAATGTCCACAAACTGA |
| *Il17a* | AGAAGATGCTGGTGGGTGTG | GGGTTTCTTAGGGGTCAGCC |
| *Cd31* | CTGCCAGTCCGAAAATGGAAC | CTTCATCCACCGGGGCTATC |
| *Epcam* | GAAGGGGCGATCCAGAACAA | TGTCCTTGTCGGTTCTTCGG |
| *Cd45* | GTTGTGCTTGGAGGGTCAGT | CTCAAACTTCTGGCCTTTGG |
| *Cnn1* | AACCCCACGACATCTTTGAG | AGCCAGGAGAGTGGACTGAA |
| *Col1a1* | TCTGACTGGAAGAGCGGAGA | GACGGCTGAGTAGGGAACAC |
| *Fn1* | ACGGTTTCCCATTACGCCAT | GGCACCATTTAGATGAATCGCA |
| *Lox* | CATCGGACTTCTTACCAAGCCG | GGCATCAAGCAGGTCATAGTGG |
| *Tgfβ1* | ATACGTCAGACATTCGGGAAGCAGTG | AATAGTTGGTATCCAGGGCTCTCCG |

**Table S2. Antibodies used for flow cytometry, immunohistochemistry and immunofluorescence.**

| **Antibodies** | **Source** | **Identifier** |
| --- | --- | --- |
| Rat monoclonal anti-CD16/32 (clone 93) | ThermoFisher Scientific | Cat# 14-0161-82; RRID:AB_467133 |
| Rat monoclonal anti-CD45-PE (clone 30-F11) | ThermoFisher Scientific | Cat# 12-0451-81;  RRID:AB_465667 |
| Rat monoclonal anti-CD11b-APC (clone M1/70) | ThermoFisher Scientific | Cat# 17-0112-82; RRID:AB_469343 |
| Rat monoclonal anti-Ly6C-eFluor 700 (clone HR1.4) | Biolegend | Cat# 128023; RRID:AB_10640119 |
| Rat monoclonal anti-Ly6G-BV421 (clone 1A8) | Biolegend | Cat# 127627; RRID:AB_127627 |
| Rat monoclonal anti-CD115-PE-Dazzle594 (clone AFS98) | Biolegend | Cat# 135527; RRID:AB:135527 |
| Rat monoclonal anti-CSF2 (clone MP122E9) | R&D Systems | Cat# MAB415-100; RRID:AB_2276702 |
| Rat polyclonal IgG | R&D Systems | Cat# 6-001-A; RRID:AB_10144734 |
| Rat monoclonal anti-CD45 (clone 30-F11) | ThermoFisher Scientific | Cat# 14-0451-82; RRID:AB_467251 |
| Mouse monoclonal anti-ERα (clone D-12) | Santa Cruz Biotechnology | Cat# sc-8005; RRID:AB_627556 |
| Rat monoclonal anti-F4/80 (clone BM8) | Biolegend | Cat# 123102; RRID:AB_893506 |
| Rabbit polyclonal anti-CD11b | Novus Biologicals | Cat# NB110-89474; RRID:AB_1216361 |
| Rat monoclonal anti-Ly6G (clone RB-6-8C5) | Abcam | Cat# ab25377; RRID:AB_470492 |
| Goat polyclonal anti-GFP | Novus Biologicals | Cat# NB100-1678; RRID:AB_10002630 |
